# Renal Tubular Epithelial TRPA1 Acts as An Oxidative Stress Sensor to Mediate Ischemia-Reperfusion-Induced Kidney Injury through MAPKs/NF-κB Signaling

**DOI:** 10.3390/ijms22052309

**Published:** 2021-02-25

**Authors:** Chung-Kuan Wu, Chia-Lin Wu, Tzong-Shyuan Lee, Yu Ru Kou, Der-Cherng Tarng

**Affiliations:** 1 Division of Nephrology, Department of Internal Medicine, Shin-Kong Wu Ho-Su Memorial Hospital, Taipei 111, Taiwan; chungkuan.wu@gmail.com; 2 School of Medicine, Fu-Jen Catholic University, New Taipei 242, Taiwan; 3 Division of Nephrology, Department of Internal Medicine, Changhua Christian Hospital, Changhua 500, Taiwan; 143843@cch.org.tw; 4 School of Medicine, Chung-Shan Medical University, Taichung 402, Taiwan; 5 Department of Physiology, College of Medicine, National Taiwan University, Taipei 106, Taiwan; ntutslee@ntu.edu.tw; 6 Department of Institute of Physiology, School of Medicine, National Yang-Ming University, Taipei 112, Taiwan; 7 Department of Biological Science and Technology, National Chiao Tung University, Hsinchu 300, Taiwan; 8 Center for Intelligent Drug Systems and Smart Bio-devices (IDS2B), Hsinchu 300, Taiwan; 9 Division of Nephrology, Department of Medicine, Taipei Veterans General Hospital, Taipei 112, Taiwan; 10 Institute of Clinical Medicine, National Yang-Ming University, Taipei 112, Taiwan

**Keywords:** acute kidney injury, TRPA1, oxidative stress, inflammation, renal tubule cells

## Abstract

Oxidative stress and inflammation play important roles in the pathophysiology of acute kidney injury (AKI). Transient receptor potential ankyrin 1 (TRPA1) is a Ca2+-permeable ion channel that is sensitive to reactive oxygen species (ROS). The role of TRPA1 in AKI remains unclear. In this study, we used human and animal studies to assess the role of renal TRPA1 in AKI and to explore the regulatory mechanism of renal TRPA1 in inflammation via in vitro experiments. TRPA1 expression increased in the renal tubular epithelia of patients with AKI. The severity of tubular injury correlated well with tubular TRPA1 or 8-hydroxy-2′-deoxyguanosine expression. In an animal model, renal ischemia-reperfusion injury (IR) increased tubular TRPA1 expression in wild-type (WT) mice. *Trpa1*^−/−^ mice displayed less IR-induced tubular injury, oxidative stress, inflammation, and dysfunction in kidneys compared with WT mice. In the in vitro model, TRPA1 expression increased in renal tubular cells under hypoxia-reoxygenation injury (H/R) conditions. We demonstrated that H/R evoked a ROS-dependent TRPA1 activation, which elevated intracellular Ca^2+^ level, increased NADPH oxidase activity, activated MAPK/NF-κB signaling, and increased IL-8. Renal tubular TRPA1 may serve as an oxidative stress sensor and a crucial regulator in the activation of signaling pathways and promote the subsequent transcriptional regulation of IL-8. These actions might be evident in mice with IR or patients with AKI.

## 1. Introduction

Renal ischemia-reperfusion injury (IR) is among the most common causes of renal dysfunction and acute kidney injury (AKI) [1,2]. Oxidative stress and inflammation play important roles in the pathophysiology of IR-induced AKI [1,2,3,4,5,6]. During the reperfusion phase of IR, excess generation of reactive oxygen species (ROS) occurs, leading to the increase in oxidative stress in renal tissues [2,3,4,5]. These ROS then initiate a complex mechanism that involves various types of cells and inflammatory mediators leading to AKI [1,2,4,6]. For example, renal tubular epithelial cells are vulnerable to oxidative stress and their injury is a key feature of the initiation phase of IR-induced AKI [7,8,9]. IR may also increase intracellular ROS to aggregate injury through the activation of NADPH oxidase [9,10,11], which is a primary enzyme system that generates ROS in mammalian cells [12]. IR induces inflammatory mediators such as interleukin-8 (IL-8), monocyte chemoattractant protein 1 (MCP-1), and macrophage inflammatory protein 2 (MIP-2), which are important chemokines that initiate and amplify renal inflammation [6,13,14,15]. These inflammatory mediators may be produced by signaling mechanisms [1], such as the redox-sensitive mitogen-activated protein kinases (MAPKs)/nuclear factor-κB (NF-κB) pathway [5,11,16,17,18]. However, the way by which renal tubular epithelial cells can sense IR-induced oxidative stress and translate these cellular events remains unclear.

Transient receptor potential ankyrin 1 (TRPA1), which is a type of nonselective transmembrane cation channel, is involved in Ca^2+^ permeability [19,20]. Neuronal TRPA1 acts as a sensor of toxic signals and molecular integrator of cellular stress, including ROS [20,21]. Recent studies have demonstrated that TRPA1 is expressed in various types of non-neuronal cells, including renal epithelial tubular cells [22,23]. Promoting inflammation is among the major functions of TRPA1 in non-neuronal cells [24]. For example, the stimulation of lung epithelial cells by cigarette smoke, which is a major oxidant, increases the TRPA1-mediated production of IL-8 [25,26]. In mice, epithelial TRPA mediates lung inflammation due to cigarette smoke [25,26]. Nevertheless, whether TRPA1 plays a role in the IR-induced inflammation and injury in the kidney remains unclear, because recent studies demonstrated that TRPA1 may protect against sepsis-induced or Ang II-induced and IR injury by modulating mitochondrial biogenesis and mitophagy or by inhibiting macrophage-mediated inflammation [27,28,29].

The aims of the study are as follows: first, to assess the correlation between the expression of renal tubular TRPA1 or 8-hydroxy-2′-deoxyguanosine (8-OHdG, a biomarker of oxidative stress) and the severity of renal tubular injury in patients with acute tubular necrosis (ATN); second, to compare renal oxidative stress, inflammation, dysfunction, and injury induced by renal IR between wild-type (WT) and *trpa1*^−/−^ mice; and finally, to investigate how renal tubular TRPA1 acts as a crucial regulator in the activation of signaling pathways and promotes IL-8 induction in renal tubular epithelial (HK-2) cells in response to hypoxia-reoxygenation injury (H/R), through an in vitro model that mimics in vivo IR [30].

## 2. Results

### 2.1. Patients with ATN and AKI Display Increased Expression of Renal Tubular TRPA1 and 8-OHdG

**Figure 1:**
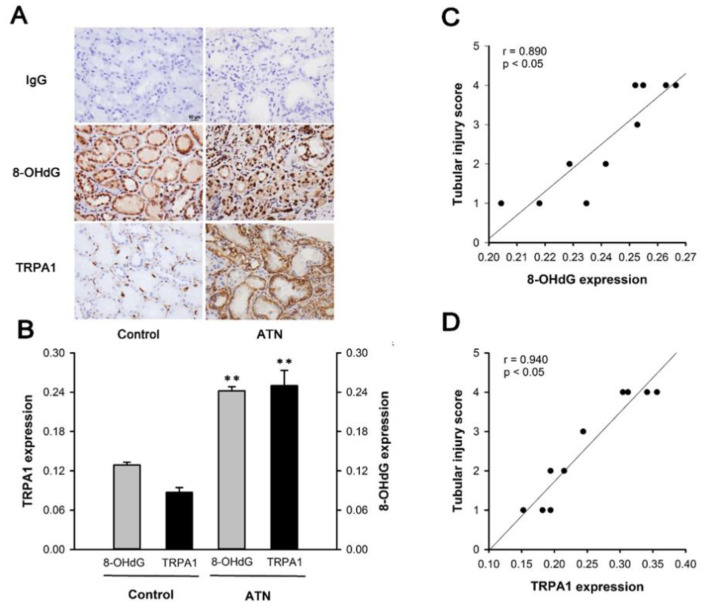
Patients with acute tubular necrosis (ATN) and acute renal injury display increased expression of renal tubular transient receptor potential ankyrin 1 (TRPA1) and 8-hydroxy-2′-deoxyguanosine (8-OHdG), a biomarker of oxidative stress. (**A**) Representative images showing immunostaining with an antibody against TRPA1 or 8-OHdG. Renal sections were obtained from normal control subjects and patients with ATN. The specificity of the immunostaining was confirmed by using an IgG-negative control. The magnification of each panel was 400×. (**B**) Quantification of the expression levels of renal tubular TRPA1 and 8-OHdG in the renal sections with immunostaining. Data in each group are the mean ± SEM from 10 patients. ** *p* < 0.01 versus the control group. (**C**,**D**) Pearson correlation analyses of expression of renal tubular TRPA1 or 8-OHdG with tubular injury score in patients with ATN (*n* = 10); r represents the correlation coefficient. Tubular injury score was calculated according to the percentage of injured area of tubular cross sections. The scoring system is described in the Methods section.

Immunohistochemical analyses showed stronger positive staining for TRPA1 or 8-OHdG in the tubular epithelia of renal sections from a patient with ATN than in that from a normal control subject (Figure 1A). Analysis of quantitative staining values revealed that the expression of renal tubular TRPA1 or 8-OHdG in the ATN group was significantly higher than in the control group (Figure 1B). Renal tubular injury scores were then evaluated by using renal sections stained via periodic acid-Schiff (PAS). Tubular injury score was highly correlated with the expression of renal tubular TRPA1 (Figure 1C; *r* = 0.89) or 8-OHdG (Figure 1D; *r* = 0.94).

### 2.2. IR Increases the Expression of TRPA1 in Renal Tubular Epithelia and Renal Tissues in WT Mice, but Not in trpa1^−/−^ Mice

**Figure 2:**
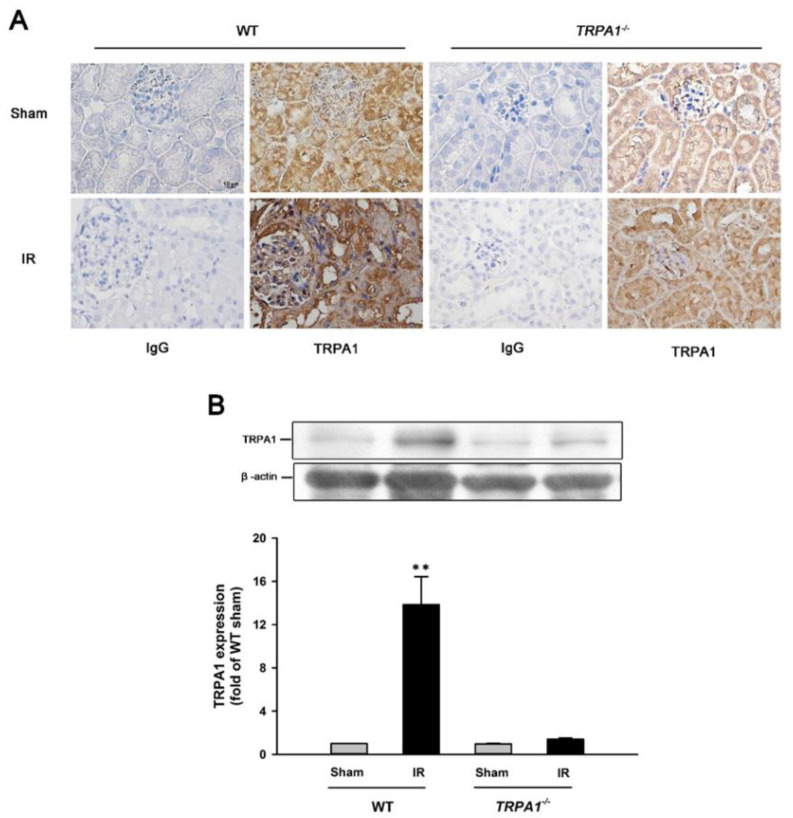
IR increases TRPA1 expression in renal tubular epithelia and renal tissues in wild type (WT) mice, but not in *trpa1*^−/−^ mice. (**A**) Representative images showing immunostaining with a TRPA1 antibody. Renal sections were obtained from two genotypes of mice subjected to two different treatments. The specificity of the immunostaining was confirmed using an IgG-negative control. The magnification of each panel was 400×. Note that the expression of tubular TRPA1 was increased by ischemia reperfusion (IR) only in WT mice. (**B**) Protein levels of TRPA1 in renal tissues obtained from the two genotypes of mice subjected to two different treatments. Protein expression was analyzed by Western blot. Immunohistochemical and Western blot analyses of renal tissues obtained from the *trpa1*^−/−^ mice displayed mild TRPA1 protein expression because they were functional knockout mice with the S5/S6 transmembrane domains of targeted gene deleted. Data in each group are mean ± SEM from 6 mice. ** *p* < 0.01 versus sham treatment.

Immunohistochemical analyses of renal sections obtained from WT mice subjected to the sham treatment revealed that their tubular epithelia displayed stronger positive staining for TRPA1 than the tubular epithelia from *trpa1*^−/−^ mice subjected to the sham treatment (Figure 2A). Additionally, the positive staining for TRPA1 in tubular epithelia from WT mice with IR was much stronger than that from WT mice subjected to the sham treatment (Figure 2A). By contrast, the positive staining for TRPA1 in tubular epithelia from *trpa1*^−/−^ mice with IR was only mildly increased compared with that from *trpa1*^−/−^ mice subjected to the sham treatment (Figure 2A). Western blot analyses using renal tissues revealed that IR significantly increased the protein expression of TRPA1 in WT mice but not in *trpa1*^−/−^ mice (Figure 2). Immunohistochemical and western blotting analyses of renal tissues obtained from the *trpa1*^−/−^ mice displayed mild TRPA1 protein expression because the *trpa1*^−/−^ mice were functional knockout mice with the S5/S6 transmembrane domains of targeted gene deleted.

### 2.3. Renal Tubular Injury Induced by IR Is Lessened in *trpa1*^−/−^ Mice

**Figure 3:**
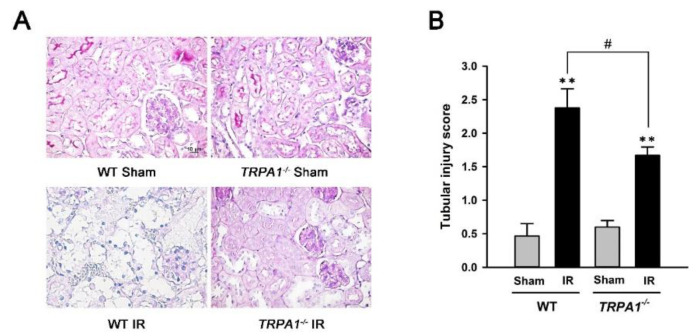
Renal tubular injury induced by IR is less in *trpa1*^−/−^ mice. (**A**) Representative images of periodic acid-Schiff (PAS) staining in renal sections obtained from the two genotypes of mice subjected to two different treatments. The magnification of each panel was 400×. (**B**) Tubular injury score was calculated according to the percentage of injured area of tubular cross sections. The scores system is described in the methods. Data in each group are mean ± SEM from 6 mice. ** *p* < 0.01 versus the sham group in each genotype; ^#^ *p* < 0.05 versus the IR group of WT mice.

Renal sections from WT mice with IR showed severe tubular injury lesions, manifested by loss of brush border, flattening, and loss of tubular epithelium and nuclear fragmentation. The extent of IR-induced changes was less in the *trpa1*^−/−^ mice (Figure 3A). Further analyses revealed that IR increased the tubular injury scores of both genotypes of mice relative to the scores of mice subjected to the sham treatment (Figure 3B). The *trpa1*^−/−^ mice with IR had significantly lower tubular injury score than the WT mice with IR (Figure 3B).

### 2.4. I/R-Induced Increase in Biomarker Levels of Renal Oxidative Stress, Inflammation, Dysfunction, and Injury Is Alleviated in Trpa1^−/−^ Mice

**Figure 4:**
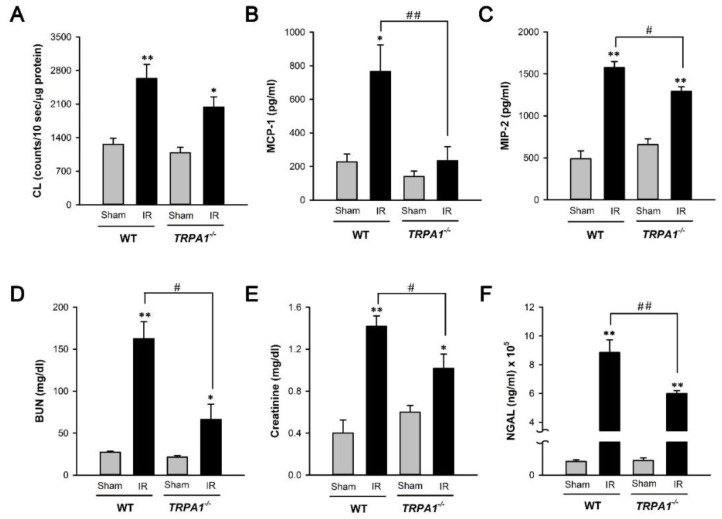
Increases in biomarker levels of renal oxidative stress, inflammation, dysfunction, and injury induced by IR are all alleviated in *trpa1*^−/−^ mice. (**A**) Quantitative data of CL counts using renal tissues for analyses. Data are presented as CL counts per 10 sec/µg protein from renal tissue lysates. (**B**) MCP-1, monocyte chemoattractant protein 1. (**C**) MIP-2, macrophage inflammatory protein 2, (**D**) BUN, blood urea nitrogen, (**E**) blood level of creatinine, (**F**) tissue levels of neutrophil gelatinase-associated lipocalin (NGAL), which is a biomarker of AKI, were analyzed by ELISA. Data in each group are mean ± SEM from 6 independent experiments. * *p* < 0.05 versus the sham group in each genotype; ** *p* < 0.01 versus the sham group in each genotype; ^#^ *p* < 0.05 versus the IR group of WT mice; ^##^ *p* < 0.01 versus the IR group of WT mice.

We compared the biomarker levels of renal oxidative stress (Figure 4A; CL-detectable superoxide anion in renal tissues), inflammation (Figure 4B,C; MCP-1 and MIP-2 in renal tissues), dysfunction (Figure 4D,E; urea nitrogen and creatinine in the blood), and kidney injury (Figure 4F; NGAL, a biomarker of kidney injury, in renal tissues) among the four study groups. IR significantly increased the levels of all biomarkers measured in both genotypes of mice compared with the levels in the mice subjected to the sham treatment (Figure 4). The levels of these biomarkers in *trpa1*^−/−^ mice with IR were significantly lower than those in WT mice with IR (Figure 4).

### 2.5. H/R Increases the TRPA1 Expression in HK-2 Cells

**Figure 5:**
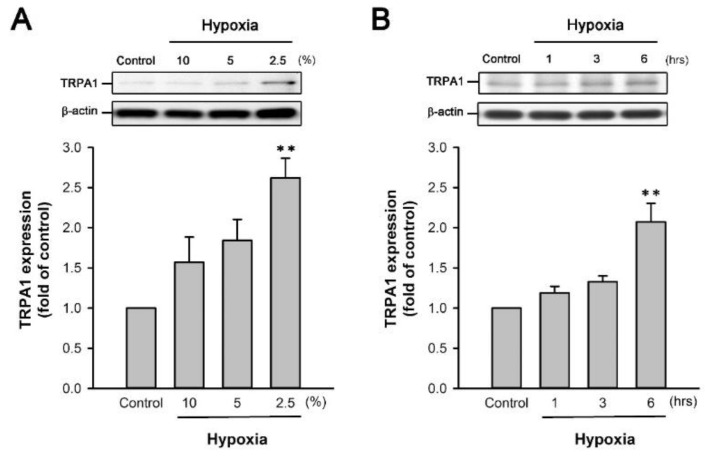
Increase in TRPA1 expression induced by H/R in HK-2 cells. (**A**) HK-2 cells were incubated under normoxia conditions as control or exposed to 2.5%, 5%, or 10% O_2_ (hypoxia) for 6 h, followed by 4 h of reoxygenation. (**B**) HK-2 cells were incubated under normoxia conditions as control or exposed to 2.5% O_2_ for 1, 3 or 6 h, followed by 4 h of reoxygenation. Protein expression was analyzed by Western blot. Data in each group are mean ± SEM from 6 independent experiments. ** *p* < 0.01 versus the control group.

The exposure of HK-2 cells to various O_2_ concentrations (10%, 5% and 2.5%) for 6 h and subsequent reoxygenation for 4 h caused concentration-dependent increase in TRPA1 protein expression (Figure 5A). Moreover, the exposure of HK-2 cells to 2.5% O_2_ for various durations (1, 3, and 6 h), followed by reoxygenation for 4 h, caused time-dependent increase in TRPA1 expression (Figure 5B). Exposure of HK-2 cells to 2.5% O_2_ for 6 h, followed by reoxygenation for 4 h, was then selected as the standard H/R conditions for the study of IL-8 induction and the signaling pathway involved.

### 2.6. TRPA1 Mediates the Induction of IL-8 by H/R in HK-2 Cells

**Figure 6:**
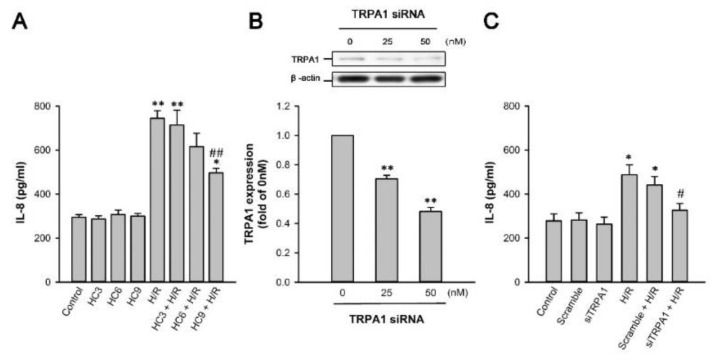
TRPA1 mediated the induction of IL-8 by H/R in HK-2 cells. (**A**) HK-2 cells were incubated under normoxia conditions as control or exposed to 2.5% O_2_ for 6 h, followed by 4 h of reoxygenation. In 6 study groups, cells were pretreated with 3, 6, or 9 µM HC-030031 (HC3, HC6 or HC 9; a TRPA1 antagonist). (**B**) HK-2 cells were incubated under normoxia conditions with or without the application of TRPA1 siRNA (siTRPA1) at two concentrations (25 and 50 nM) 48 h prior to the measurement of TRPA1 expression. Protein expression was analyzed by Western blot. (**C**) HK-2 cells were incubated under normoxia conditions as control or exposed to 2.5% O_2_ for 6 h, followed by 4 h of reoxygenation. In the 4 study groups, cells were pretreated with 50 nM of siTRPA1 or scramble siRNA. Protein expression was analyzed by ELISA. Data in each group are mean ± SEM from 6 independent experiments. * *p* < 0.05 versus the control group; ** *p* < 0.01 versus the control group; ^#^ *p* < 0.05 versus the H/R group without pretreatment of siRNA; ^##^ *p* < 0.01 versus the H/R group without pretreatment of HC.

The pretreatment of HK-2 cells with 3, 6, or 9 µM HC-030031 (a TRPA1 antagonist) dose-dependently attenuated H/R-induced IL-8 but did not affect the basal IL-8 concentrations in cells without H/R (Figure 6A). Pretreatment with TRPA1 small interfering RNA (siRNA) for gene knockdown significantly reduced TRPA1 proteins that were available for activation (Figure 6B). Additionally, pretreatment with TRPA1 siRNA (50 nM) significantly reduced H/R-induced increase in IL-8 production, whereas pretreatment with scramble siRNA failed to produce such effects (Figure 6C).

### 2.7. H/R Causes ROS-Dependent, TRPA1-Mediated Increases in Intracellular Ca2+ and NADPH Oxidase Activity in HK-2 Cells

**Figure 7:**
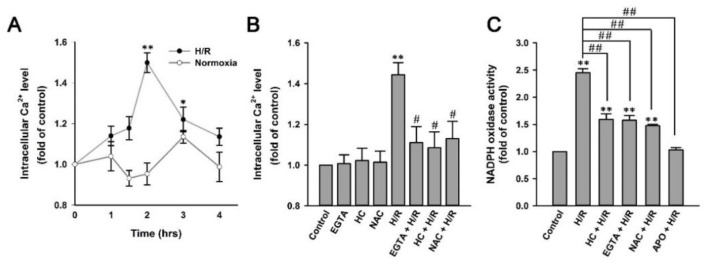
H/R causes ROS-dependent, TRPA1-mediated increases in intracellular Ca^2+^ and NADPH oxidase activity in HK-2 cells. (**A**) HK-2 cells were incubated under normoxia conditions or exposed to 2.5% O_2_ for 6 h, followed by 1, 1.5, 2, 3, and 4 h of reoxygenation. (**B**) HK-2 cells were incubated under normoxia conditions as control or exposed to 2.5% O_2_ for 6 h, followed by 2 h of reoxygenation. In 6 study groups, cells were pretreated with EGTA (an extracellular Ca^2+^ chelator; 500 µM), HC-030031 (HC, a TRPA1 antagonist; 9 µM) or N-acetyl-cysteine (NAC, a scavenger of ROS; 1 mM). (**C**) HK-2 cells were incubated under normoxia conditions as control or exposed to 2.5% O_2_ for 6 h, followed by 2 h of reoxygenation. In 4 study groups, cells were pretreated with HC-030031, EGTA, NAC or apocynin (APO; an inhibitor of NADPH oxidase; 150 µM). Intracellular Ca^2+^ levels were measured by Fluo-8 fluorescent probe assay. NADPH oxidase activity was measured by NADP^+^/NADPH assay. Data in each group are mean ± SEM from 5 independent experiments. ** *p* < 0.01 versus the control group or time zero; ^#^ *p* < 0.05 versus the H/R group without drug pretreatment; ^##^ *p* < 0.01 versus the H/R group without drug pretreatment.

After the exposure of HK-2 cells to 2.5% O_2_ for 6 h, the increase in intracellular Ca^2+^ level peaked at 2 h of reoxygenation and declined at 3 and 4 h of reoxygenation. Nevertheless, at 3 h, the level remained higher than the baseline level (Figure 7A). By contrast, the exposure of HK-2 cells to normoxia for the same duration did not significantly change the intracellular Ca^2+^ level (Figure 7B). Pretreatment with an extracellular Ca^2+^ chelator (EGTA), HC-030031 (a TRPA1 antagonist), or N-acetyl-cysteine (NAC, a ROS scavenger) greatly prevented H/R-induced increases in the intracellular Ca^2+^ level, which was measured after 2 h of reoxygenation but did not affect the basal intracellular Ca^2+^ level in cells without H/R (Figure 7B). H/R also increased NADPH oxidase activity at 2 h of reoxygenation (Figure 7C). Pretreatment with EGTA, HC-030031, NAC, or apocynin (an NADPH oxidase inhibitor) greatly prevented such an increase (Figure 7C).

### 2.8. H/R-Induced Intracellular ROS Increase via a ROS-Dependent, TRPA1-Mediated, and NOX-Released Pathway in HK-2 Cells

**Figure 8:**
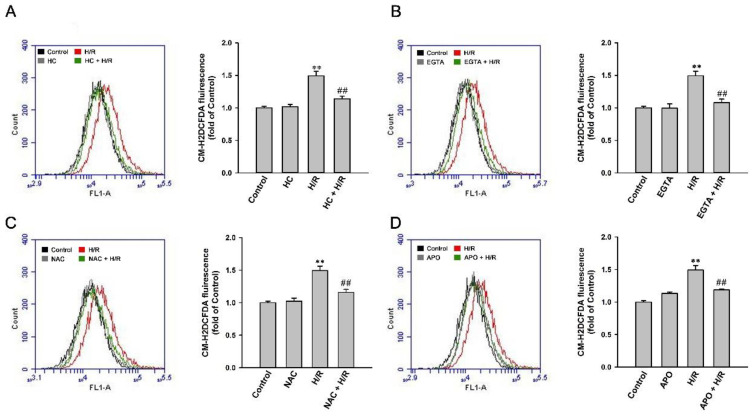
H/R-induced intracellular ROS increase via a ROS-dependent, TRPA1-mediated, and NOX-released pathway in HK-2 cells. (**A**) HK-2 cells were incubated under normoxia conditions (control) or exposed to 2.5% O_2_ for 6 h, followed by 4 h of reoxygenation. In two study groups, cells were pretreated with HC-030031 (HC, a TRPA1 antagonist; 9 µM). (**B**) HK-2 cells were incubated under normoxia conditions (control) or exposed to 2.5% O_2_ for 6 h, followed by 4 h of reoxygenation. In the two study groups, cells were pretreated with EGTA (an extracellular Ca^2+^ chelator; 500 µM). (**C**) HK-2 cells were incubated under normoxia conditions (control) or exposed to 2.5% O_2_ for 6 h, followed by 4 h of reoxygenation. In two study groups, cells were pretreated with N-acetyl-cysteine (NAC, a scavenger of ROS; 1 mM). (**D**) HK-2 cells were incubated under normoxia conditions (control) or exposed to 2.5% O_2_ for 6 h, followed by 4 h of reoxygenation. In two study groups, cells were pretreated with apocynin (APO; an inhibitor of NADPH oxidase; 150 µM). Intracellular ROS levels were measured by flow cytometry, and CM-H2DCFDA (general oxidative stress indicator) was used for the assay. Data in each group are the mean ± SEM from five independent experiments. ** *p* < 0.01 versus the control group or time zero; ^##^ *p* < 0.01 versus the H/R group without drug pretreatment.

After the exposure of HK-2 cells to 2.5% O_2_ for 6 h, an increase in intracellular ROS level at 4 h of reoxygenation was observed (Figure 8). Pretreatment with HC-030031 (a TRPA1 antagonist; Figure 8A), an extracellular Ca^2+^ chelator (EGTA; Figure 8B), apocynin (an NADPH oxidase inhibitor; Figure 8C), or N-acetyl-cysteine (NAC, a ROS scavenger; Figure 8D) greatly prevented an H/R-induced increase in the intracellular ROS level after 4 h of reoxygenation but did not affect the basal intracellular ROS level in cells not exposed to H/R.

### 2.9. MAPKs/NF-κB Pathway Is Vital for the TRPA1-Mediated Induction of IL-8 by H/R in HK-2 Cells

**Figure 9:**
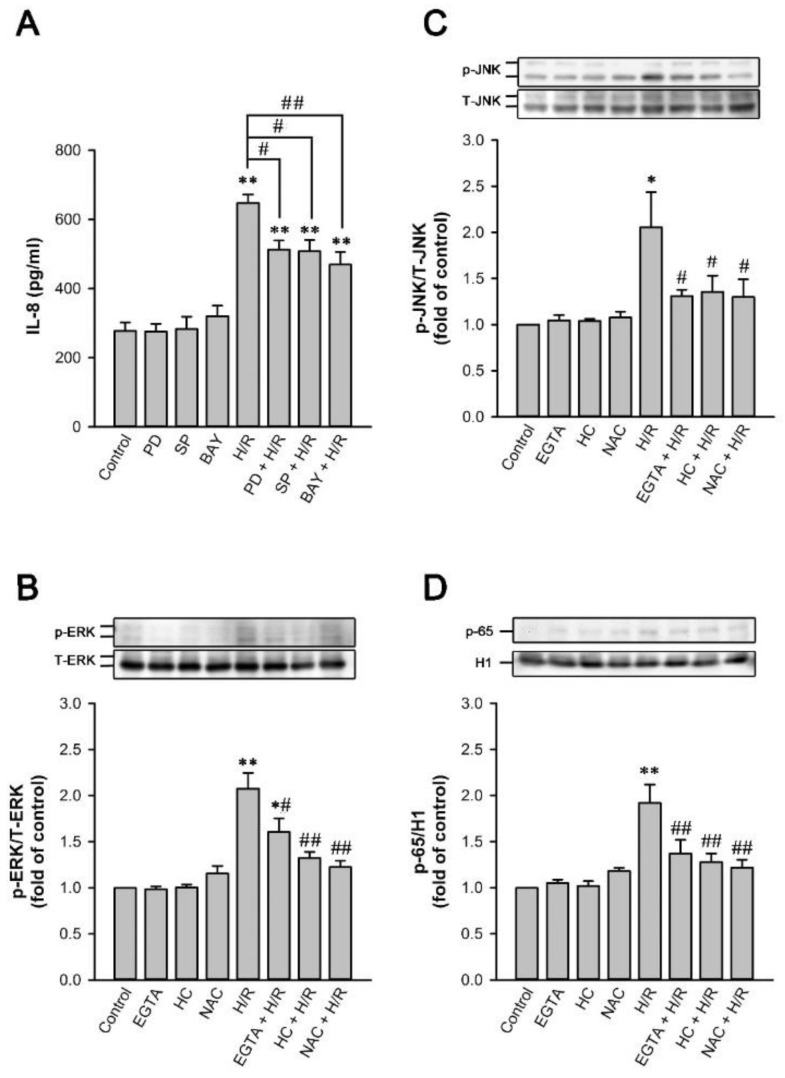
ERK/JNK/NF-*κ*B pathway is vital for the TRPA1-mediated induction of IL-8 by H/R in HK-2 cells. In all panels HK-2 cells were incubated under normoxia conditions as control or exposed to 2.5% O_2_ for 6 h, followed by 4 h of reoxygenation. (**A**) In 6 study groups, cells were pretreated with PD98059 (PD, an ERK inhibitor; 10 µM), SP600125 (SP, a JNK inhibitor; 10 µM) or BAY11-7085 (BAY, a NF-*κ*B inhibitor; 10 µM). (**B**–**D**) In 6 study groups, cells were pretreated with EGTA, HC-030031 (HC) or N-acetyl-cysteine (NAC). Protein expression was analyzed by ELISA (**A**) or Western blot (**B**–**D**). Activation of ERK and JNK was indicated by increased phosphorylation of these kinases, whereas NF-*κ*B activation was indicated by the increased presence of p65 subunit in the nucleus. Data in each group are as the mean ± SEM from 5 independent experiments. * *p* < 0.05 versus the control group; ** *p* < 0.01 versus the control group; ^#^ *p* < 0.05 versus the H/R group without drug pretreatment; ^##^ *p* < 0.01 versus the H/R group without drug pretreatment. ERK, extracellular signal–regulated kinase; JNK, c-Jun N-terminal kinase; NF-*κ*B, nuclear factor *κ*-light-chain-enhancer of activated B cells; H1, histone protein 1, served as the loading control for nuclear protein fractions.

Pretreatment of HK-2 cells with an inhibitor of extracellular signal-regulated kinases (ERK) (PD98059), an inhibitor of c-Jun N-terminal kinases (c-JNK) (SP600125), or an NF-κB inhibitor (BAY11-7085) significantly reduced the induction of IL-8 by H/R (Figure 9A). Moreover, compared with control cells, the exposure of HK-2 cells to H/R increased the amounts of phosphorylated ERK (Figure 9B), phosphorylated JNK (Figure 9C), and NF-κB p65 subunit in the cell nuclei (Figure 9D). The H/R-induced activation of MAPKs/NF-κB signaling was significantly attenuated by pretreatment with EGTA, HC-030031, or NAC (Figure 9B–D).

## 3. Discussion

A strong correlation between the renal tubular injury and the expression of renal tubular TRPA1 or oxidative stress was demonstrated for the first time in patients with ATN and AKI (Figure 1). These observations led us to hypothesize that renal tubular TRPA1 plays a detrimental role in the pathogenesis of IR-induced AKI. We used an in vivo model with IR [8,15,16,31] and an in vitro model with H/R [5,30] to investigate the detrimental function of renal tubular TRPA1 and the underlying mechanism.

Our in vivo study results showed that, compared with the WT mice, the *trpa1*^−/−^ mice displayed reduced levels of IR-induced inflammation and injury in the kidney. The levels were identified based on the alleviation of increased indices of oxidative stress, inflammation, dysfunction, and injury in the kidney (Figure 3 and Figure 4). The vulnerability of renal tubular cells to oxidative stress makes their responses a key feature in the initiation of IR-induced AKI [7,8,9]. Therefore, we believe that the reduced renal inflammation and injury observed in our *trpa1*^−/−^ mice is, at least in part, due to the absence of tubular epithelial TRPA1 functions in the *trpa1*^−/−^ mice. Additionally, other cell types, such as endothelial cells, macrophages, and leukocytes, may participate in the development of IR-induced AKI [13,32]. Accordingly, the lack of TRPA1 function in these cells may be related to IR-induced inflammation and injury in the kidney in *trpa1*^−/−^ mice. In addition, the correlation between macrophage movement and the function of TRPA1 channel [33,34,35] possibly existed in the kidney, because MCP-1 (a chemoattractant chemokine), and MIP-2 (secreted by macrophages) were reduced in *trpa1*^−/−^ mice.

Intriguingly, the IR-induced increase in the ROS level of renal tissues was lower in the *trpa1*^−/−^ mice compared with the WT mice (Figure 4). At least two sources may contribute to the overall level of tissue ROS. Infiltrated inflammatory cells, such as macrophages and neutrophils, are among the major sources of ROS in AKI [7,11]. Given that IR-induced renal inflammation was alleviated in *trpa1*^−/−^ mice, the reduced ROS level in renal tissues observed in *trpa1*^−/−^ mice may be due to the reduction of ROS from these sources. Renal tubular TRPA1 possibly plays inflammatory and injurious roles in the pathogenesis of IR-induced AKI, and these detrimental functions may be linked to IR-induced oxidative stress. However, Ma et al. [29] demonstrated TRPA1 may play a protective role in IR-induced AKI through inhibiting the classical activation of macrophages, especially M1 macrophages. Their results are contradictory to the present results from animal study. One explanation may be because the TRPA1 in renal tubular epithelium and macrophages might exert differential effects after IR. Eliminating the TRPA1 function in any cell leads to variable effects on IR. For the same reason, the role and mechanism of TRPA1 in myocardial IR are conflicting and remain controversial. Conklein et al. [36] stated that in mouse model of IR, the global knockout of TRPA1 results in less myocardial injury. However, Lu et al. [37] reported that the administration of the TRPA1 agonist reduced the myocardial infarct size in myocardial IR, and the activation of the TRPA1 channel during reoxygenation in vitro decreased cardiomyocyte cell death and the release of lactate dehydrogenase. Therefore, a genetically modified mouse model with cell-specific deletion of TRPA1 in kidney is needed to assess the role of TRPA1 in renal IR. Additionally, the use of different renal IR models or *trpa1*^−/−^ mice might also explain the difference in results.

Results of our in vitro study showed that the exposure of HK-2 cells to H/R caused several events. First, H/R increased IL-8 production, which can be attenuated by antagonizing TRPA1 with HC-030031 or gene silencing targeting for TRPA1 (Figure 6), suggesting the detrimental role of TRPA1 in this IL-8 induction. We consistently observed an increase in the expression of renal tubular TRPA1 in patients with AKI (Figure 1A), mice with IR (Figure 2A), and HK-2 cells with H/R (Figure 5); thus, the detrimental functions of renal tubular TRPA1 may be augmented by this increased expression. Second, H/R increased the intracellular level of Ca^2+^ at a time point prior to the IL-8 induction in HK-2 cells (Figure 7). This elevated intracellular level of Ca^2+^ clearly demonstrated the ROS-dependent activation of TRPA1 by H/R, because it could be totally prevented by EGTA, HC-030031, or NAC. TRPA1 is well known for its function in Ca^2+^ permeability [19,20]. Moreover, ROS may cause the oxidation of disulfide bonds near the pore region of these channels or produce the covalent modification of cysteines within electrophile/oxidant-sensing sites; both functions lead to the activation of TRPA1 [38,39]. NAC is membrane-permeable [40]; hence, it presumably can scavenge extracellular and intracellular ROS, both of which were possibly responsible for the activation of renal tubular TRPA1 in this study.

Third, H/R increased NADPH oxidase activity in HK-2 cells. This activity was partially suppressed by EGTA, HC-030031, or NAC and was totally abolished by apocynin (Figure 7). These findings suggest that the H/R-induced activation of NADPH oxidase was at least in part possibly due to the increase in intracellular Ca^2+^ after the ROS-dependent activation of TRPA1. These in vitro data suggested the vital role of NADPH oxidase in the mechanism of increased oxidative stress in IR-related AKI, as proposed by other investigators [41]. As demonstrated in our patient study, the level of renal tubular injury correlated well with the level of oxidative stress in patients with ATN and AKI (Figure 1). Thus, we speculated that the renal tissues of our patients with AKI may exhibit the overexpression of NADPH oxidase. Indeed, a clinical study reported that the expression of p22^phox^ subunit of NADPH oxidase was significantly greater in patients with AKI with poor outcomes compared with those with favorable outcomes [42]. Our findings are in agreement with those of previous studies showing that the TRPA1- or stress-mediated elevation of the intracellular Ca^2+^ concentration acts as an upstream signal for the activation of NADPH oxidase [26,43]. Thus, the activation of NADPH oxidase has been known to increase the oxidative stress induced by IR in vivo [9,10,11] and to increase intracellular ROS induced by H/R in vitro [17,44]. Additionally, a crosstalk exists between the ROS-dependent activation of TRPA1 and the TRPA1/Ca^2+^-mediated production of ROS. H/R increased intracellular ROS level in HK-2 cells. The increase was suppressed by HC-030031, EGTA, apocynin, or NAC (Figure 8). The H/R-induced intracellular ROS probably increased via a ROS-dependent, TRPA1-mediated, and NOX-released pathway in HK-2 cells. Indeed, a similar interplay is suggested in many cell types [39,45]. In these cells, Ca^2+^ signaling mediated by various Ca^2+^ channels can regulate NADPH oxidase activity, thereby increasing intracellular ROS, which can regulate the activity of these Ca^2+^ channels in a reciprocal manner [32]. This crosstalk was present in our in vitro model.

Lastly, H/R activated the MAPK/NF-κB signaling in HK-2 cells (Figure 9). Using pharmacological inhibitors, we proved that this signaling was involved in the induction of IL-8 by H/R (Figure 9A). The activation of the MAPK/NF-κB signaling was dependent on the functions of TRPA1, Ca^2+^, and ROS because it was prevented by HC-030031, EGTA, or NAC (Figure 9). Previous studies showed that the activation of this signaling pathway contributes to the pathogenesis of IR-induced AKI [5,11,16,17,18]. Intracellular Ca^2+^ [26,46] and ROS [5,11,17,26] are potential triggers for the activation of this signaling. We further characterized this pathway. It is a process that relies on the ROS-dependent activation of TRPA1. This finding provided additional evidence that increased our understanding of the cellular events involved in the transcription regulation of IL-8 by H/R in renal tubular cells.

The present study had several limitations. First, the mouse model of unilateral renal IR with contralateral nephrectomy was chosen because of the high mortality rate of mice after bilateral renal IR in our animal experiment, even if the AKI model is more relevant to human pathological conditions. Second, the primary cultured cells from kidneys after IR cannot survive for a long time under our experimental conditions, as the cells are damaged in the oxidative challenges. Therefore, we chose HK-2, which is an immortalized proximal tubule epithelial cell line from normal adult human kidney, for the in vitro molecular study. Third, the measurement of 4-hydroxynonenal (4-HNE), an important oxidative stress maker and an agonist of TRPA1, was not performed in our in vivo and in vitro study.

Currently, TRPA1 is not the only type of TRP channels that purportedly to participates in the regulation of renal physiology and pathology. A recent review article by Ma et al. [47] summarized the evidence that TRP channel, subfamily C, and member 6 (TRPC6) expressed in several types of renal cells are involved in renal injury in animal models and human subjects. Ma et al. also showed that TRPC6 is ROS-sensitive, and its regulatory role in Ca^2+^ signaling is vital to oxidative stress-related kidney diseases [47]. Wu et al. [48] showed that the knockout or pharmacological inhibition of TRPC6 in mice ameliorates renal fibrosis induced by unilateral ureteral obstruction. Gao et al. [49] reported that TRP channel, subfamily M, member 2 (TRPM2) is localized mainly in the kidney proximal tubular epithelial cells of mice. Similar to our findings, the knockout or pharmacological inhibition of TRPM2 in mice attenuates IR-induced inflammation, injury, elevated oxidative stress, and increased NADPH oxidase activity in the kidney [50]. Chen et al. [51] reported that the activation of transient receptor potential vanilloid 1 (TRPV1) by agonists ameliorates IR-induced AKI in rats, but these channels are localized in the sensory nerves distributed in the kidney.

## 4. Materials and methods

### 4.1. Reagents

Antibodies, kits and reagents used are described in Supplemental Tables S1 and S2. 

### 4.2. Human Study 

A total of 10 adult patients with AKI and biopsy-proven ATN at Changhua Christian Hospital, Taiwan who were not kidney transplant recipients and did not have active malignancy, were recruited. They met the Acute Kidney Injury Network criteria [52], but AKI was not pre-renal or had obstructive etiology. The clinical information of the biopsy-proven ATN patients is listed in Supplemental Table S3. For the control group, 10 adult patients who had normal kidney functions were enrolled; they received nephrectomy for localized circumscribed renal tumors. Normal renal tissues were obtained from the uninvolved poles of removed kidneys. The study was approved by the Institutional Review Board of Changhua Christian Hospital (approval number 150912; approval date Nov 14, 2015).

### 4.3. Animals and Renal Ischemia-Reperfusion Injury Model

Male *trpa1*^−/−^ mice (Jackson Laboratory, ME, USA), which had the S5/S6 transmembrane domains of the targeted gene deleted, were functional knockout mice. Male *trpa1*^−/−^ mice and WT C57BL/6 mice (National Laboratory Animal Center, Taipei, Taiwan) were housed in individual cages under specific pathogen-free conditions in an air-conditioned animal facility and were exposed to a 12 h/12 h light/dark cycle. The mice had free access to water and food.

The method to perform renal IR was described previously [31]. Renal IR was performed in 8–12 week-old mice 20–25 g in weight under deep anesthesia. Anesthesia treatment was conducted by using MatrxTM VIP 3000 Vaporizer with isoflurane. The right kidney was removed from each mouse after clamping the renal pedicle. After 1 week, the left kidney was exposed and a microvascular clip was used to clamp the pedicle of the left kidney for 30 min as renal ischemia. The clamp was removed, and the surgical wound was closed after the color of left kidney changed from purple to deep red. After 24 h of reperfusion, the mice were euthanized. Kidneys from age-matched and sham-operated mice served as controls.

### 4.4. Cell Culture and H/R Model

HK-2 cells were cultured under standard cell culture conditions (37 °C, 5% CO_2_, 21% O_2_, and 74% N_2_) in Dulbecco’s modified Eagle’s medium (DMEM, Corning Mediatech, Manassas, VA, USA) containing 4.5 g/L glucose, L-glutamine, and sodium pyruvate and supplemented with 10% fetal bovine serum (FBS, Thermo Fisher Scientific, Waltham, MA, USA) and 1% 100× penicillin-streptomycin solution. The method used to perform H/R was described previously [5,29]. For the hypoxic treatment, HK-2 cells were incubated in DMEM with 5% FBS in a hypoxic chamber containing various concentration of oxygenation for different durations. After hypoxia, the cells were maintained under standard cell culture conditions and subjected to reoxygenation for different durations (0–4 h). HK-2 cells cultured in DMEM under normoxic conditions were used as control.

### 4.5. Histopathology and Tubular Injury Score

Formalin-fixed and paraffin-embedded human and experimental murine kidney tissues were sectioned at 3 µm thickness and stained for histological examination. The sections were stained with a periodic acid-Schiff (PAS) staining kit according to the manufacturer’s instructions. Twenty randomly selected sections were assessed. Tubular injury (tubular cell swelling, loss of brush border, or nuclear condensation) was scored from 0 to 4 (0, no change; 1, changes affecting 25% of the section; 2, changes affecting 25% to 50%; 3, changes affecting 50% to 75%; and 4, changes affecting 75% to 100%) as previously described [6,51].

### 4.6. Immunohistochemistry 

Immunohistochemistry (IHC) of human and murine renal tissues was performed with previously described methods [5]. Rabbit Ab against TRPA1 or mouse Ab against 8-OHdG was used. Immunoglobulin G (IgG) Ab was used as the negative control. An evaluation of IHC staining was performed by computer-assisted pixel counts (Image-Pro Plus 6.0, Media Cybernetics, Silver Spring, MD, USA). Twenty areas from each IHC sample were randomly selected in the renal cortex and examined under a microscope, and the images were captured with an Olympus Microscope BX51 (Olympus, Tokyo, Japan). Quantitative immunohistochemical staining value was calculated as the integrated optical density divided by the total area occupied by the DAB-stained and hematoxylin-stained cells of each slide.

### 4.7. Serum BUN and Creatinine

Before operation and at the time of euthanasia, blood samples were collected from the facial veins of the mice and placed into an Eppendorf tube to measure the serum BUN and creatinine. The measurements were performed with the reagent strips of Arkray Spotchem TMII and Spotchem EZ SP-4430 (Arkray, Minneapolis, MN, USA).

### 4.8. Tissue Superoxide Production

Superoxide counts were measured using a previously described method [5] with minor modifications. Murine renal tissue was homogenized and chemiluminescence (CL) was measured continuously with an Analyzing System (CLD-110, Tohoku Electronic Industry Co., Sendai, Japan). Total CL was calculated by integrating the area under the curve and subtracting it from the background level. The assay was performed in duplicate for each sample and normalized by using the total protein weight. Data were expressed as CL count/10 s/μg protein.

### 4.9. Inflammatory Chemokines and NGAL

The concentrations of pro-inflammatory chemokines, MCP-1, MIP-2, neutrophil gelatinase-associated lipocalin (NGAL) from mouse renal tissues, and IL-8 from the medium under different conditions were measured using ELISA kits (MCP-1, PeproTech, Rocky Hill, NJ, USA), (MIP-2, MyBioSource, San Deigo, CA, USA), (NGAL, Bioporto, Hellerup, Denmark), (IL-8, Koma Biotech, Seoul, South Korea) according to the manufacturer’s instructions.

### 4.10. Western Blot

Cell pellets or murine renal tissue were homogenized in 80–100 μL of RIPA lysis buffer; the protein amount was measured using a BCA protein assay kit (Pierce Biochemical, Rockford, IL, USA). For the in vitro study, nuclear extracts were prepared using a previous method [53]. An equal amount of protein was separated on 10% sodium dodecyl sulfate–polyacrylamide gel by electrophoresis (SDS-PAGE) and then transblotted onto a PVDF membrane. After being blocked with T-pro fast blocking buffer, the blots were incubated with primary Abs and then with corresponding secondary Abs. The protein bands were detected with an ECL kit and then quantified by Image J (1.50i, National Institutes of Health, Bethesda, MD, USA). In the animal study, kidney tissues from the renal IR of *trpa1*^−/−^ and WT mice and from the sham operation of *trpa1*^−/−^ and WT mice were analysed by Western blot. In the in vitro study, HK-2 cells were incubated under normoxia or exposed to different O_2_ concentrations for 6 h, followed by 4 h reoxygenation or different times of 2.5% O_2_ and finally 4 h of reoxygenation. HK-2 cells were incubated under normoxia with or without the application of TRPA1 siRNA (siTRPA1) at two concentrations (25 and 50 nM) for 48 h prior to the measurement of TRPA1 expression. HK-2 cells pre-treated with EGTA, HC-030031 (HC) or N-acetyl-cysteine (NAC) were also incubated under normoxia or exposed to 2.5% O_2_ for 6 h, followed by 4 h of reoxygenation to investigate the MAPK/ NF-κB signalling pathway. The protein expression in these in vitro studies were analysed by Western blot. HK-2 cells were exposed to all pre-treated reagents for 1 h prior to hypoxia or normoxia treatment.

### 4.11. siRNA Transfection

HK-2 cells were seeded in 6-well plates overnight. Then, the transient transfection of scramble siRNA (non-targeting negative control) or siRNAs against human TRPA1 (ON-TARGET plus Human TRPA1 siRNA, Dharmacon, CO, USA) was performed for 48 h using DharmaFECT transfection reagents according to the manufacturer’s instructions. TRPA1 expression was detected by Western blot analysis. In some experiments, transiently transfected cells were subjected to H/R treatment, and IL-8 expression was detected. The sequences of siRNAs against TRPA1 are listed in the Supplemental Table S4.

### 4.12. Intracellular Ca2+ Levels and NADPH Oxidase Activity

Intracellular Ca^2+^ levels were determined using a Screen QuestTM Fluo-8 Medium Removal Calcium Assay Kit (AAT Bioquest, Inc., Sunnyvale, CA, USA), which is based on fluorescence intensity. The cells were cultured in growth medium overnight. Afterward, the medium was replaced by Fluo-8 NW dye-loading solution, a calcium indicator that can reflect the intracellular calcium level. Motion fluorescence intensity at Ex/Em = 490/525 nm was determined after 1 h of incubation at room temperature. NADPH oxidase activity was examined using an EnzyChromTM NADP^+^/NADPH assay kit (BioAssay Systems, Hayward, CA, USA), which is based on a glucose dehydrogenase cycling reaction in which the formed NADPH reduces a formazan reagent. The intensity of the reduced product colour at 565 nm is proportional to the NADP^+^/NADPH concentration and reflects the NADPH oxidase activity in the tested samples.

### 4.13. Detection of ROS by Flow Cytometry

ROS generation was measured using the CM-H2DCFDA fluorescence dye (Thermo Fisher Scientific, Waltham, MA, USA). To detect ROS, the HK-2 cells were pretreated with 9 µM HC-030031, 500 µM EGTA, 150 µM APO, or 1 mM NAC for 1 h before transferring to a hypoxic chamber containing 2.5% oxygen for 6 h, followed by reoxygenation for 4 h. Subsequently, cells were washed twice with PBS, detached with Accutase (Corning Mediatech, Manassas, VA, USA), and resuspended in 1 mL of PBS, and 1.5 μL of 0.5 mM CM-H2DCFDA. The cells were gently mixed and incubated for 15 min at 37 °C in the dark. Next, the cells were rewashed and suspended in 300 μL of cold PBS. Finally, the ROS generation concentrations were monitored using a flow cytometer (Accuri C6, BD Biosciences, Mississauga, ON, Canada), and data were processed with the Accuri C6 software (BD Biosciences). The fluorescence intensity in cells was determined by integrating the area under the curve of the spectrum using flow cytometry. The average of the fluorescence intensity in HK-2 cells under normoxic conditions was used as the control.

### 4.14. Statistical Analysis

The results are shown as mean ± standard error of the mean (SEM). Statistical analysis was performed by one-way analysis of variance followed by Dunnett’s test or Fisher’s least significant difference for multiple comparisons as appropriate. The association between two variables was analyzed by using Pearson’s correlation. Differences were considered statistically significant at *P* < 0.05.

## 5. Conclusions

**Figure 10:**
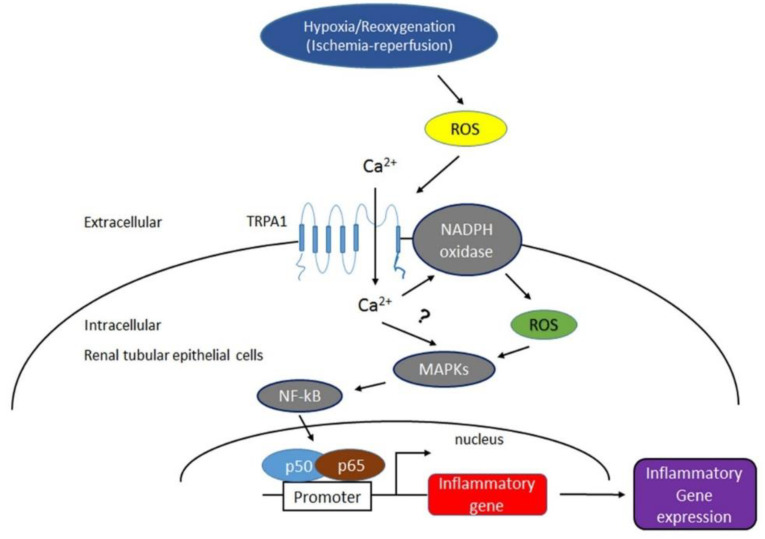
The proposed mechanism of TRPA1 activation in renal tubular epithelial cells by hypoxia-reoxygenation (H/R) or I/R for the induction of renal inflammation and renal injury. As shown, H/R or I/R causes ROS increase, which in turn activates renal epithelial TRPA1, thereby, ultimately promoting of Ca^2+^ influx. The increase of intracellular Ca^2+^ in renal epithelial cells then contributes to the activation of NADPH oxidase, which can result in the elevation of the intracellular ROS. This effect activates the MAPKs/NF-κB signaling pathway, which allows the induction of inflammatory chemokines and aggravates renal injury.

In summary, results from our patient study suggested that the expression of renal tubular TRPA1 is well correlated with renal tubular injury or oxidative stress in patients with ATN and AKI. Our in vivo findings indicated that renal tubular TRPA1 plays a detrimental role in IR-induced AKI. Our in vitro findings suggested that the ROS-dependent activation of TRPA1 is responsible for the increased intracellular Ca^2+^, the increased NADPH oxidase activity, the activation of the MAPK/NF-κB signaling, and the induction of IL-8 in renal tubular cells with H/R. Thus, renal tubular TRPA1 may act as an oxidative stress sensor and a crucial regulator in the activation of signaling pathways and may promote the subsequent transcriptional regulation of IL-8. This cellular mechanism may possibly be at work in patients with AKI or mice with IR (Figure 10). Our findings provided significant novel information about the pathogenic mechanisms associated with IR-induced AKI and contributed to the development of potential therapies.

## Data Availability

The data presented in this study are available on request from the corresponding author. The data are not publicly available due to legal restrictions imposed by the government of Taiwan in relation to the “Personal Information Protection Art”.

## Supplementary Materials

The following are available online at https://www.mdpi.com/1422-0067/22/5/2309/s1.
