# Supplementary material for: Renal Tubular Epithelial TRPA1 Acts as An Oxidative Stress Sensor to Mediate Ischemia-Reperfusion-Induced Kidney Injury through MAPKs/NF-κB Signaling
Source: Int J Mol Sci. 2021 Feb 25;22(5):2309. doi: 10.3390/ijms22052309 (PMC7956664; doi:10.3390/ijms22052309)
Supplement: Supplementary file 1 [file ijms-22-02309-s001.pdf]

Supplementary files

Renal tubular epithelial TRPA1 acts as an oxidative stress sensor to mediate ischemia-reperfusion-induced kidney injury through MAPKs/NF-κB signaling

Chung-Kuan Wu[1,2], Chia-Lin Wu[3,4], Tzong-Shyuan Lee[5], Yu-Ru Kou[6*] and Der-Cherng Tarng[6,7,8,9*]

[1] Division of Nephrology, Department of Internal Medicine, Shin-Kong Wu Ho-Su Memorial Hospital, Taipei 111, Taiwan; chungkuan.wu@gmail.com

[2] School of Medicine, Fu-Jen Catholic University, New Taipei 242, Taiwan; chungkuan.wu@gmail.com

[3] Division of Nephrology, Department of Internal Medicine, Changhua Christian Hospital, Changhua 500, Taiwan; 143843@cch.org.tw

[4] School of Medicine, Chung-Shan Medical University, Taichung 402, Taiwan; 143843@cch.org.tw

[5] Department of Physiology, College of Medicine, National Taiwan University, Taipei 106, Taiwan; ntutslee@ntu.edu.tw

[6] Department of Institue of Physiology, School of Medicine, National Yang-Ming University, Taipei 112, Taiwan; yrkou@ym.edu.tw (Y.R.K.); dctarng@vghtpe.gov.tw (D.-C.T.)

[7] Department of Biological Science and Technology, National Chiao Tung University, Hsinchu 300, Taiwan; and Center for Intelligent Drug Systems and Smart Bio-devices (IDS2B), Hsinchu 300, Taiwan; dctarng@vghtpe.gov.tw

[8] Division of Nephrology, Department of Medicine, Taipei Veterans General Hospital, Taipei 112, Taiwan; dctarng@vghtpe.gov.tw

[9] Institute of Clinical Medicine, National Yang-Ming University, Taipei 112, Taiwan; dctarng@vghtpe.gov.tw

* Correspondence: yrkou@ym.edu.tw; Tel: +886-2-2867086; Fax: +886-2-2864049 and dctarng@vghtpe.gov.tw; Tel: +886-2-28757517; Fax: +886-2-28757841

Table S1. Antibodies used for Western blot, and immunohistochemical staining; kits used for enzyme linked immunosorbent assay

Target	Figure	Source	App
TRPA1	1, 2, 5, 6	Rabbit Ab, Novus Biologicals (Littleton, CO, USA)	IHC, WB
8-OHdG	1	Mouse Ab, Abcam (Cambridge, UK)	IHC
β-actin	2, 5, 6	Mouse Ab, Proteintech Group (Chicago, IL, USA)	WB
ERK	9	Rabbit Ab, Proteintech Group (Chicago, IL, USA)	WB
c-JNK			
P65			
H1			
Phospho-ERK	9	EnoGene Biotech (New York, NY, USA)	WB
Phospho-JNK			
Mouse NGAL	4	Bioporto (Hellerup, Denmark)	ELISA
Mouse MCP-1	4	PeproTech (Rocky Hill, NJ, USA)	ELISA
Mouse MIP-2	4	MyBioSource (San Deigo, CA,USA)	ELISA
Human IL-8	6,9	Koma Biotech (Seoul, South Korea)	ELISA

Abbreviations: TRPA1, transient receptor potential ankyrin 1; 8-OHdG, 8-hydroxy-2-deoxyguanosine; ERK, extracellular signal-regulated kinases; c-JNK, c-Jun N-terminal kinases; P65, a subunit of NF-*k*B transcription complex; H1, histone protein 1; NGAL, neutrophil gelatinase-associated lipocalin; MCP-1, monocyte chemoattractant protein 1; MIP-2, macrophage inflammatory protein 2; IL-8, interleukin-8; Ab, antibody; App, application; WB, Western blot; IHC, immunohistochemical staining; ELISA, enzyme linked immunosorbent assay

Table S2. List of reagents, vehicles used to dilute the tested drugs, and companies

Reagents	Vehicles	Companies
EGTA	NaOH	Sigma Aldrich (Merck KGaA, Darmstadt, Germany)
NAC	H_2O	
Apocynin	DMSO	
BAY11-7085		
HC-030031		Cayman Chemical (Ann Arbor, MI, USA)
PD98059		Calbiochem (Merck KGaA, Darmstadt, Germany)
SP600125		
DMEM		Corning Mediatech, Manassas, VA, USA
Penicillin-streptomycin solution		
FBS		Thermo Fisher Scientific, Waltham, MA, USA
PAS staining kit		Merck Millipore, Billerica, MA, USA
siRNA transfection reagent		GE Healthcare Life Sciences

Abbreviations: EGTA, ethylene glycol-tetraacetic acid; NAC, N-acetyl-cysteine; DMEM, Dulbecco's Modified Eagle Medium; FBS, fetal bovine serum; PAS, periodic acid-Schiff; DMSO, dimethyl sulfoxide; N/A, not available.

Table S3. The clinical information of the biopsy-proven ATN patients.

No	Age	Gender	DM	HTN	AKIN	TIS	TRPA1 expression	8-OHdG expression
1	73.8	1	0	0	3	3	0.244	0.253
2	70.3	1	1	1	3	4	0.356	0.267
3	35.5	1	0	0	3	4	0.341	0.263
4	35.2	1	0	0	3	4	0.312	0.255
5	19.4	0	0	0	2	4	0.312	0.252
6	69.6	1	1	0	3	2	0.215	0.229
7	73.4	0	0	1	1	2	0.194	0.242
8	72.3	1	0	1	3	1	0.194	0.218
9	58.9	0	0	0	1	1	0.182	0.235
10	56.0	1	1	0	2	1	0.152	0.204
**Data**	56.4 ±19.6	7 (70%)	7 (70%)	3 (30%)	2.4 ±0.8	2.6 ±1.3	0.250 ±0.073	0.241 ±0.020

The codes of binary variables are that gender (male=1, female=0), DM (patients with history of type 2 DM=1, patients without type 2 DM=0), HTN (patients with history of HTN=1, patients without HTN=0), respectively. AKIN: Acute kidney injury network for classification of severity of acute kidney injury (AKI); TIS: tubular injury score; Data are expressed as *n* (%) for categorical data and as mean ± standard deviation for continuous data in the Data Row.

Table S4. List of the siTRPA1 component

Name	Target Sequences (sense)	Antisense
D-006109-01	GAAGGACGCUCUCCACUUA	UAAGUGGAGAGCGUCCUUC
D-006109-02	GGACAAUGGUGCACAAAUA	UAUUUGUGCACCAUUGUCC
D-006109-03	GGCAAUAAAUGUGCAAUUA	UAAUUGCACAUUUAUUGCC
D-006109-04	UCAAAGAGCUGGUAAUGGA	UCCAUUACCAGCUCUUUGA
